# Ethidium Bromide Modifies The Agarose Electrophoretic Mobility of CAG•CTG Alternative DNA Structures Generated by PCR

**DOI:** 10.3389/fncel.2017.00153

**Published:** 2017-05-30

**Authors:** Mário Gomes-Pereira, Darren G. Monckton

**Affiliations:** ^1^Laboratory CTGDM, INSERM UMR1163Paris, France; ^2^Institut Imagine, Université Paris Descartes—Sorbonne Paris CitéParis, France; ^3^Institute of Molecular, Cell and Systems Biology, College of Medical, Veterinary and Life Sciences, University of GlasgowGlasgow, United Kingdom

**Keywords:** trinucleotide DNA repeat, myotonic dystrophy, non-B DNA, ethidium bromide, PCR, agarose, polyacrylamide, electrophoresis

## Abstract

The abnormal expansion of unstable simple sequence DNA repeats can cause human disease through a variety of mechanisms, including gene loss-of-function, toxic gain-of-function of the encoded protein and toxicity of the repeat-containing RNA transcript. Disease-associated unstable DNA repeats display unusual biophysical properties, including the ability to adopt non-B-DNA structures. CAG•CTG trinucleotide sequences, in particular, have been most extensively studied and they can fold into slipped-stranded DNA structures, which have been proposed as mutation intermediates in repeat size expansion. Here, we describe a simple assay to detect unusual DNA structures generated by PCR amplification, based on their slow electrophoretic migration in agarose and on the effects of ethidium bromide on the mobility of structural isoforms through agarose gels. Notably, the inclusion of ethidium bromide in agarose gels and running buffer eliminates the detection of additional slow-migrating DNA species, which are detected in the absence of the intercalating dye and may be incorrectly classified as mutant alleles with larger than actual expansion sizes. Denaturing and re-annealing experiments confirmed the slipped-stranded nature of the additional DNA species observed in agarose gels. Thus, we have shown that genuine non-B-DNA conformations are generated during standard PCR amplification of CAG•CTG sequences and detected by agarose gel electrophoresis. In contrast, ethidium bromide does not change the multi-band electrophoretic profiles of repeat-containing PCR products through native polyacrylamide gels. These data have implications for the analysis of trinucleotide repeat DNA and possibly other types of unstable repetitive DNA sequences by standard agarose gel electrophoresis in diagnostic and research protocols. We suggest that proper sizing of CAG•CTG PCR products in agarose gels should be performed in the presence of ethidium bromide.

## Introduction

The expansion of unstable trinucleotide repeat DNA sequences is the genetic cause of severe human disorders, including myotonic dystrophy type 1 (DM1), Huntington disease (HD), Friedreich ataxia (FRDA) and fragile X syndrome (FRAXA; Pearson et al., 2005; Gomes-Pereira and Monckton, 2006; Zhao and Usdin, 2015). At least 15 of these conditions, including DM1, HD and many spinocerebellar ataxias (SCAs), have been associated with the expansion of CAG•CTG triplet repeats (Gomes-Pereira and Monckton, 2006; Zhao and Usdin, 2015). The downstream pathogenesis triggered by the repeat expansions varies between diseases and can be mediated by different molecular mechanisms (Gatchel and Zoghbi, 2005). Among these, RNA toxicity was first described in DM1 (Mankodi et al., 2000), a multisystemic disorder caused by the abnormal expansion of a non-coding CAG•CTG trinucleotide DNA repeat (Brook et al., 1992). In addition to DM1, toxic RNA transcripts have been implicated in the pathogenesis of other CAG•CTG expansion diseases, such as SCA type 8 (SCA8), SCA12, HD, Huntington disease like-2 (HDL2) and Fuchs endothelial corneal dystrophy (FECD; Sicot et al., 2011; Sicot and Gomes-Pereira, 2013; Du et al., 2015).

Disease-associated CAG•CTG trinucleotide repeats are dramatically unstable in the germline and in somatic cells, exhibiting a marked tendency towards further expansion. Since longer repeats are associated with a more severe phenotype and an earlier age of onset, expansion-biased germline repeat instability accounts for the phenomenon of anticipation; whereby an earlier age of onset and more severe symptoms are observed in successive generations. In addition, mounting evidence suggests that tissue-specific, age-dependent, expansion-biased somatic mosaicism most likely contributes towards the tissue-specific symptoms and disease progression in CAG•CTG expansion disorders (Kennedy et al., 2003; Wheeler et al., 2003; Swami et al., 2009; Morales et al., 2012). As a result, deciphering the molecular mechanisms of trinucleotide repeat expansion will not only help understand important aspects of disease pathogenesis, but it may also open new avenues to the development of novel therapeutic interventions (Gomes-Pereira and Monckton, 2006; López Castel et al., 2010).

A number of different molecular mechanisms of trinucleotide repeat expansion have been suggested, involving aberrant DNA replication, DNA repair and/or DNA homologous recombination (Richards and Sutherland, 1994; Richard and Pâques, 2000; Pearson et al., 2005; Gomes-Pereira and Monckton, 2006; Mirkin, 2007; McMurray, 2010; Usdin et al., 2015). Although dependent on different molecular events, most of the proposed mechanisms share a unifying theme: the speculation that repeat-specific non-B-DNA structures adopted by either or both of the DNA strands are involved in the mechanism of genetic instability, acting as the driving force for expansion (Sinden and Wells, 1992; McMurray, 1999; Wells et al., 2005; Mirkin, 2006; Usdin et al., 2015). Computational studies have predicted unusual biophysical properties of trinucleotide repeat sequences (Baldi et al., 1999). CAG•CTG trinucleotide repeats in particular, form stable intrastrand DNA hairpins, not only *in vitro* (Gacy et al., 1995) but also in mammalian cell models of CAG•CTG instability (Liu et al., 2010). Most interestingly, a novel form of double-stranded non-B-DNA structure has been identified. Melting and re-annealing of plasmid DNA or gene fragments containing CAG•CTG repeats results in a high proportion of the DNA population adopting alternative conformations with retarded mobility in native polyacrylamide gels (Pearson and Sinden, 1996). Biochemical evidence and electron microscopy data are consistent with the existence of slipped-stranded DNA (S-DNA) structures formed within the triplet repeats in otherwise linear duplex molecules (Pearson et al., 1997, 1998, 2002; Tam et al., 2003). Both the propensity for S-DNA formation and the complexity of the structures increase with the length of the pure repeat tract (Pearson and Sinden, 1996; Pearson et al., 1997) and parallels the probability of repeat expansion, suggesting a critical role in the mechanism of repeat size mutation. Indeed, the levels of slipped-stranded DNA structures isolated by immunoprecipitation from different DM1 patient tissues correlate with the degree of somatic mosaicism (Axford et al., 2013). Such structures are recognized and bound by the mismatch repair protein MSH2 (Pearson et al., 1997), possibly to promote their retention and subsequent repeat expansion (Kovtun and McMurray, 2001; Savouret et al., 2004; Tomé et al., 2009; Guo et al., 2016). In addition to functional MSH2, at least four other components of the DNA mismatch repair pathway are necessary to mediate high levels of CAG•CTG somatic expansion (Manley et al., 1999; van Den Broek et al., 2002; Gomes-Pereira et al., 2004; Foiry et al., 2006; Seriola et al., 2011; Pinto et al., 2013; Tomé et al., 2013; Morales et al., 2016). Moreover, sequence interruptions within pure repeat tracts dramatically decrease the propensity to form slipped-stranded DNA structures as well as the heterogeneity of the structures formed (Pearson et al., 1998). The protective effect of sequence interruptions on the formation of alternative CAG•CTG DNA structures parallels a stabilizing effect in DM1 (Musova et al., 2009; Braida et al., 2010; Botta et al., 2017), SCA1 (Chung et al., 1993; Chong et al., 1995), SCA2 (Choudhry et al., 2001) and SCA17 (Gao et al., 2008). Taken together these observations strongly suggest a role of slipped-strand structures in the process of CAG•CTG repeat expansion.

The DNA intercalating dye ethidium bromide interacts directly with DNA, and is capable of altering sequence-dependent structural features, such as handedness, planarity and twisting of curved DNA segments (Brukner et al., 1997; Hayashi and Harada, 2007; Lipfert et al., 2010). Ethidium bromide is therefore likely to alter structural features of alternative trinucleotide repeat sequences when added to agarose gels. We have investigated the implications of this intercalating dye on the conformational metabolism of CAG•CTG repetitive sequences, and developed an easy method to detect the presence of alternative non-B-DNA structures based on the differential electrophoretic profiles of trinucleotide repeat sequences in agarose gels in the presence and absence of ethidium bromide.

## Materials and Methods

### PCR Amplification and Analysis

Single sized DM1 alleles containing 5, 22, 44, 56 or 200 CTG repeat units were isolated by serial dilution and single molecule PCR amplification of human genomic DNA, as previously described (Gomes-Pereira et al., 2004). These alleles were then used as input DNA (0.1–1 ng) in subsequent standard PCR amplifications, using the previously described oligonucleotide primers DM-A, DM-BR, DM-C, DM-DR, DM-ER, DM-H (Monckton et al., 1995, 1997). PCR amplifications, agarose gel electrophoresis, Southern “squash” blotting and hybridization were performed as previously described (Gomes-Pereira et al., 2004). The AmpliSize^TM^ molecular ruler (50–2000 bp ladder; BioRad) or the GeneRuller 100 bp ladder (Thermo Scientific), and the Kodal Digital Science 1D software (Kodak) were used to quantify electrophoretic mobility and DNA sizing. All participants in the study were recruited with informed consent for genetic studies in myotonic dystrophy.

### Non-Denaturing Polyacrylamide Gel Electrophoresis

Non-denaturing polyacrylamide gel electrophoresis (PAGE) was carried out in a BioRad Protean II gel apparatus, using 8% (w/v) non-denaturing acrylamide/bis (29:1) gels, containing 10% (v/v) glycerol, in 1X TBE (90 mM Trizma base, 90 mM orthoboric acid, 2 mM EDTA). The polymerizing agents *NNN’N’-*tetramethylethylenediamine (TEMED) and ammonium persulfate (APS) were added to a final concentration of 0.625% (v/v) and 0.125% (w/v), respectively. Prior to loading, the gels were equilibrated by applying 5 Vcm^−1^ for 90 min with continuous re-circulation of 1X TBE running buffer at 4°C. DNA samples were resolved at 10 Vcm^−1^ for 16 h at 4°C with buffer re-circulation. The gel was stained with 500 nM ethidium bromide in 1X TBE for 20 min at room temperature. Separated DNA samples were visualized using a UV transilluminator (wavelength 254 nm) and photographed. Alternatively, DNA was transferred from the polyacrylamide gel onto a nylon membrane by Southern “squash” blotting, and hybridized with a radiolabeled CAG•CTG-containing probe, as previously described (Gomes-Pereira et al., 2004). The AmpliSize^TM^ molecular ruler (50–2000 bp ladder; BioRad) and the Kodal Digital Science 1D software (Kodak) were used to quantify electrophoretic mobility and DNA sizing.

### DNA Extraction from Non-Denaturing Polyacrylamide Gels

Bands corresponding to the DNA samples of interest were excised from the polyacrylamide gel using a scalpel under UV transillumination. DNA was eluted from the gel fragments by simple diffusion, in 500 μl of 1X TE (10 mM Tris•HCl pH 8.0, 1 mM EDTA), at 37°C for 48 h. Linear polyacrylamide was used as a DNA carrier to precipitate gel-purified DNA samples, as previously described (Gaillard and Strauss, 1990).

### Denaturing and Re-Annealing Protocols

DNA species eluted from non-denaturing PAGE were either heat-denatured or subjected to a re-annealing protocol. The denaturation procedure consisted of heating the DNA at 100°C for 5 min in the presence of 40% (v/v) formamide, followed by rapid chilling on ice. The re-annealing protocol included an initial melting step at 100°C for 5 min, followed by slow cooling to 18°C, at an average rate of −0.5°C per minute. Melting and cooling was performed in a Biometra Uno thermal cycler.

## Results

### Altered Electrophoretic Mobility of Trinucleotide Repeat Sequences in the Presence of Ethidium Bromide

**Figure 1:**
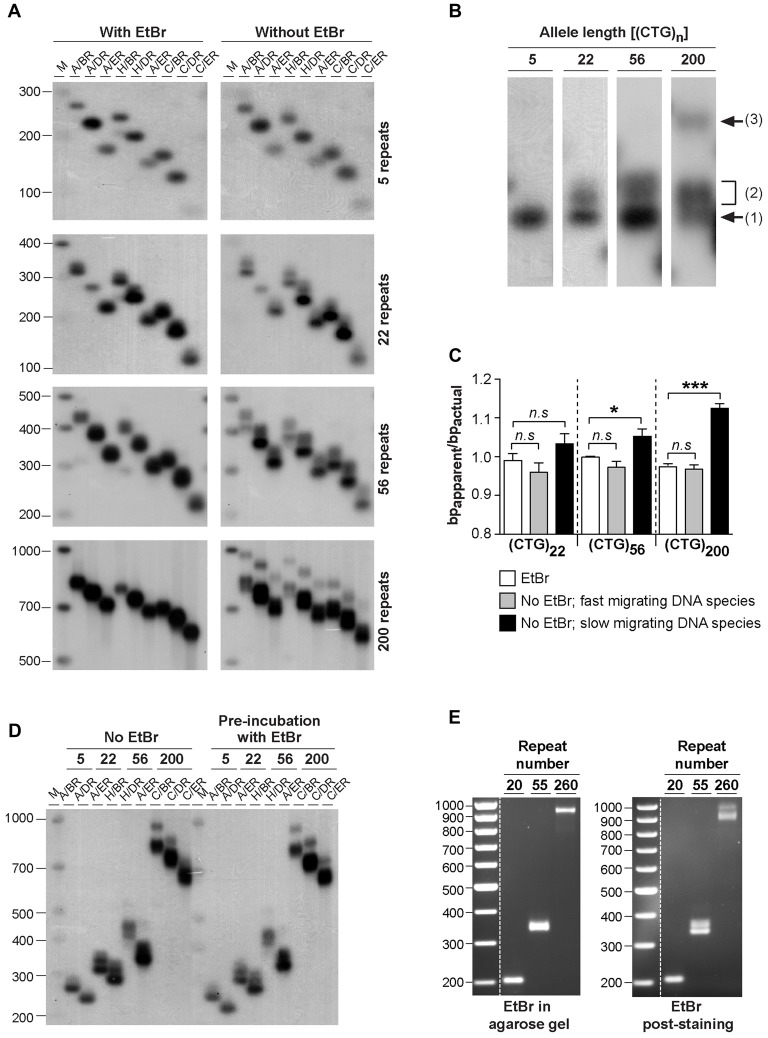
**Effect of ethidium bromide on the mobility of CAG•CTG sequences in agarose gels. (A)** The presence/absence of ethidium bromide modifies the mobility of PCR amplified expanded CAG•CTG repeats during agarose gel electrophoresis. PCR products containing 5, 22, 56 or 200 CAG•CTG repeats were individually isolated from the human *DM1* locus by serial dilution, subsequently amplified with various combinations of DM-specific oligonucleotide primers (DM-A, DM-H, DM-C, DM-BR, DM-DR and DM-ER) and resolved through 1.8% (w/v) agarose gels, with (left) or without (right) 500 nM ethidium bromide in both the gel and 1X TBE running buffer. The amplified products were subsequently detected by Southern “squash” blot hybridization. The autoradiographs illustrate the increased mobility of alternative expanded CAG•CTG conformers in agarose gels in the presence of ethidium bromide. Note the multiple alternative products observed in the absence of the intercalating chemical. The scale on the left represents the position and sizes of the molecular weight markers in bp (M). **(B)** The absence of ethidium bromide in the gel and running buffer results in the detection of additional slow migrating species during agarose gel electrophoresis of expanded CAG•CTG sequences. The autoradiograph shows a close-up of DM1 PCR products resolved in an agarose gel in the absence of ethidium bromide. Note the position of the putative B-DNA structures (1) and non-B-DNA structures (2 and 3). **(C)** The intense fast-migrating bands observed in the absence of ethidium bromide exhibited the same electrophoretic mobility as the single band detected when ethidium bromide was present. The ratio between the relative mobility in agarose gel and the actual size (bp_apparent_/bp_actual_) of the DNA species is shown for PCR products containing 22, 56 and 200 repeats. The graph shows a significant difference between the electrophoretic mobility of the slow migrating DNA species detected in the absence of ethidium bromide and the single band detected in the presence of the dye (*p < 0.05; ***p < 0.001; one-way analysis of variance (ANOVA)). **(D)** Pre-incubation with ethidium bromide fails to eliminate additional slow migrating species during agarose gel electrophoresis of PCR-amplified expanded CAG•CTG repeats. DM1 alleles with different repeat numbers were amplified by PCR using multiple oligonucleotide primer combinations, indicated in the figure above each lane. Half of each PCR product was incubated with 500 nM of ethidium bromide for over 48 h prior to electrophoresis and hybridization analysis. The autoradiographs do not reveal any detectable difference in the electrophoretic profile between treated and non-treated samples. **(E)** Ethidium bromide modifies the agarose electrophoretic mobility of CAG•CTG products generated by bulk PCR amplification of 20–100 ng of template genomic DNA. Ethidium bromide post-staining revealed slow migrating DNA species containing 56 and 200 CAG•CTG repeats, which were undetected when the dye was added to the gel and running buffer.

In order to investigate the possible effect of ethidium bromide on the biophysical properties of trinucleotide repeat double-stranded DNA sequences, we have analyzed the electrophoretic profiles of CAG•CTG repetitive sequences derived from the *DM1* locus under different conditions. DNA sequences, containing a different number of CAG•CTG repeats (ranging from 5 up to 200), were generated by two rounds of PCR amplification, both involving <30 cycles. Human genomic DNA was initially amplified by single molecule small pool-PCR (SP-PCR), with oligonucleotide primers DM-A and DM-BR (Monckton et al., 1995). Separated PCR products containing different sized CAG•CTG tracts were subsequently amplified by standard PCR with various oligonucleotide primer combinations, to generate different sized flanking regions. The samples were resolved through 1.8% (w/v) agarose gels, either with or without 500 nM ethidium bromide, in both the gel and electrophoresis buffer, and detected by Southern “squash” blot hybridization (Gomes-Pereira et al., 2004). In the presence of ethidium bromide, PCR products migrated predominantly as a single major DNA species, and generated in most cases a sharp and well defined band (Figure 1A). In contrast, the same amplification products containing 22, 56 and 200 triplet repeats generated one or two additional slow-migrating bands in the absence of ethidium bromide, for all the flanking sequences studied (Figures 1A,B). The PCR products containing only five repeat units did not produce multiple bands under the same conditions. The 22 repeat PCR products generated a single additional slow migrating species. The 55 repeat PCR products generated a single, but very broad additional DNA band, that might represent a diffuse doublet. The longest PCR products containing 200 CTG repeats generated two clearly separable slow-migrating species (bands 2 and 3, Figure 1B). A similar effect of ethidium bromide on the electrophoretic mobility of expanded triplet repeat sequences through agarose gels was observed for PCR products derived from the HD, SCA7, *FECD/CTG18.1* and *ERDA1* loci (Braida et al., unpublished observations), indicating that this is a general phenomenon for CAG•CTG repeat sequences.

**Figure 2:**
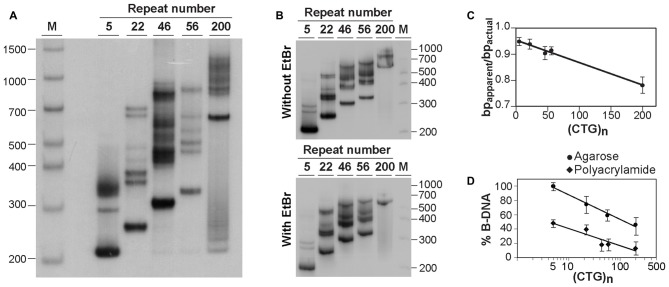
**Analysis of alternative DNA structures of CAG•CTG-containing PCR products by native polyacrylamide gel electrophoresis (PAGE). (A)** Detection of multiple slow migrating species during native PAGE of PCR amplified expanded CAG•CTG repeats. Different length DM1 alleles were amplified using oligonucleotide primers DM-A and DM-DR, and resolved through a non-denaturing 8% (w/v) polyacrylamide gel, containing 10% (v/v) glycerol in the absence of ethidium bromide. The PCR products were detected by Southern blot hybridization. The autoradiograph shows the presence of a complex pattern of multiple bands for each PCR product. **(B)** The same PCR products containing a varying number of CAG•CTG repeats were resolved by native PAGE in the absence (top) or presence (bottom) of 500 nM ethidium bromide in the gel and running buffer. Ethidium bromide does not affect the detection of slow migrating DNA species in non-denaturing polyacrylamide gels. The molecular size markers in bp (M) are shown. **(C)** Longer CAG•CTG repeat products migrate aberrantly fast during native PAGE. The ratio between the apparent size of native species in native polyacrylamide and their actual size (bp_apparent_/bp_actual_) is plotted against the repeat number of the PCR products. The graph shows a linear relationship (*r*^2^ = 0.986, *p* < 0.001), indicating that the increase in the electrophoretic mobility through non-denaturing polyacrylamide gels increases as the CAG•CTG repeat tract lengthens. **(D)** The proportion of non-B-DNA species present in the PCR amplification products of CAG•CTG repeat sequences increases with allele length. The percentage of B-DNA detected in agarose and non-denaturing polyacrylamide gels was estimated by densitometric analysis for each PCR product, and plotted as a function of the CAG•CTG repeat number (log scale). The non-linear regression indicates a decrease in the percentage of duplex B-DNA detected in agarose (*r*^2^ = 0.989, *p* = 0.03) or in polyacrylamide gels (*r*^2^ = 0.855, *p* = 0.02) as the repeat gets longer.

To quantify the effect of ethidium bromide on the electrophoretic mobility of PCR-generated CAG•CTG sequences in agarose gels, we have calculated the ratio between the apparent size of DNA species and the actual sequence size (*R* = bp_apparent_/bp_actual_), as previously described (Chastain et al., 1995). This figure serves as an estimate of the aberrant DNA migration relative to a known molecular size marker. We focused on repeat sizes that generated slow migrating bands (22, 56 and 200 CAG•CTG repeats), and found that the bp_apparent_/bp_actual_ ratio of the fast migrating DNA species (detected in the absence of ethidium bromide) was not significantly different from that of the single band detected in the presence of ethidium bromide. In contrast, slow migrating DNA species displayed a significantly higher bp_apparent_/bp_actual_ ratio, showing increases of ~5% and ~11% for PCR products containing 56 or 200 CAG•CTG repeats, respectively (Figure 1C). Given the identical electrophoretic mobility of the fast-migrating bands in the absence of ethidium bromide and the single band detected when the intercalating dye was added to the gel and running buffer, we assumed that the former represent putative linear B-DNA molecules. Moreover, densitometric quantification of putative non-B-DNA species detected in this assay revealed that the relative amount of fast-migrating B-DNA molecules decreased with longer alleles (Figure 2D). In other words, the electrophoretic profile of the amplification products in the absence of ethidium bromide becomes more complex as the repeat sequence lengthens.

To determine if the intercalating properties of ethidium bromide *per se* were responsible for this effect, selected PCR products were incubated with 500 nM ethidium bromide for 48 h prior to electrophoresis. Under these conditions, agarose gel electrophoresis without ethidium bromide added to the gel or buffer resulted in the detection of the same additional slow-migrating bands as observed in the complete absence of ethidium bromide at any stage (Figure 1D). These data suggest that ethidium bromide is only capable of altering the mobility of structural isomers formed within CAG•CTG expanded sequences when present in the gel and running buffer, and does not appear to mediate direct interconversion of structural isoforms.

To exclude the possibility of confounding structural artifacts introduced by multiple rounds of PCR amplification, we have analyzed repeat sequences generated by direct amplification of 10–100 ng genomic DNA from DM1 transgenic mice carrying 20, 55 or 260 CAG•CTG repeats within a human *DMPK* transgene (Gourdon et al., 1997; Seznec et al., 2000). The resolution of PCR products through agarose gels containing 500 nM of ethidium bromide yielded single sharp bands of the expected size. In contrast, post-staining of ethidium bromide-free gels revealed slow migrating DNA species for repeat sizes of 55 CAG•CTG and above (Figure 1E). These results demonstrate that ethidium bromide generally affects the electrophoretic mobility of CAG•CTG repeat sequences generated by PCR amplification protocols.

### Detection of DNA Alternative Structures of CAG•CTG Sequences in Native Polyacrylamide Gel Electrophoresis

To confirm the presence of alternative non-B-DNA structures in the PCR products, the sequences amplified with oligonucleotide primers DM-A and DM-DR were resolved through a native 8% (w/v) polyacrylamide gel, with 10% (v/v) glycerol, and detected by Southern “squash” blot hybridization. Although each PCR product generated a single band following agarose gel electrophoresis in the presence of ethidium bromide (Figure 1A), complex electrophoretic profiles, consisting of an intense fast migrating band and a series of novel, closely-spaced DNA species, were observed on native polyacrylamide gels (Figure 2A). Interestingly, the presence of ethidium bromide in the gel and/or running buffer did not greatly affect the mobility of the alternative DNA sequences through native polyacrylamide gels (Figure 2B). The patterns of anomalously migrating products were very similar to those previously described for triplet repeat-containing gene fragments or plasmids following denaturation and renaturation protocols (Pearson and Sinden, 1996; Pearson et al., 1998).

As reported previously, the major band detected for each PCR product migrated faster than expected for the putative double-stranded B-DNA molecule through native polyacrylamide gels (Chastain et al., 1995; Pearson and Sinden, 1996; Pearson et al., 1998). To quantify this effect and estimate the aberrant DNA migration relative to known molecular size markers, we have used the bp_apparent_/bp_actual_ ratio for each PCR product under native PAGE, as described (Chastain et al., 1995). For the major fast migrating band, the ratio varied from 0.95 for the PCR product containing five CTG repeats (corresponding to an ~5% increase in mobility) down to 0.78 for the longest repeat (corresponding to an ~22% increase in mobility). Overall, repeat length accounts for ~99% of the variation in bp_apparent_/bp_actual_ ratios (*r^2^* = 0.986, *p* < 0.001; Figure 2C), suggesting that the fast mobility of these sequences in native polyacrylamide gels is intimately dependent on the repeat size, and is probably associated with the enhanced flexibility of the trinucleotide repeat tract as previously suggested (Chastain and Sinden, 1998). The additional lower mobility bands defined electrophoretic patterns that were similar for all triplet repeat lengths analyzed. The slowly migrating products were characterized by a wide range of size ratios (bp_apparent_/bp_actual_), varying from ~1.3 to ~2.6, probably representing discrete DNA isoforms (Table 1). It is noteworthy that the slow migrating band derived from the shortest PCR product (5 CAG•CTG repeats) displayed a size ratio bp_apparent_/bp_actual_ of 1.56, whereas longer alleles (22, 46 and 56 repeats) gave rise to bands with size ratios up to ~2.5. This observation may indicate that the corresponding alternative structure cannot be formed with such a low repeat number. The longest amplification product (200 CAG•CTG) produced a heterogeneous pattern, and it was therefore difficult to accurately define the bands. The slight differences between the ratios reported here, and the ratios described by other authors, may be attributed to the mobility dependence on the length and nature of DNA sequences flanking the triplet repeat (Tam et al., 2003).

**Table 1:** **Relative mobilities of CAG•CTG-containing PCR products through native polyacrylamide gels**.

	5 CAG•CTG		22 CAG•CTG		46 CAG•CTG		56 CAG•CTG		200 CAG•CTG	
Band	bp_app_	bp_app_/bp_actual_	bp_app_	bp_app_/bp_actual_	bp_app_	bp_app_/bp_actual_	bp_app_	bp_app_/bp_actual_	bp_app_	bp_app_/bp_actual_
1	214	0.95	260	0.94	315	0.90	346	0.91	633	0.78
2	297	1.31	373	1.35	465	1.33	478	1.26	1048	1.29
3	352	1.56	403	1.45	534	1.53	517	1.37		
4			447	1.62	602	1.72	572	1.51		
5			494	1.78	837	2.40	639	1.68		
6			640	2.31			894	2.36		
7			708	2.56						

*The table displays the apparent size of individual bands relative of each PCR product, relative to a known molecular ruler, as determined by native PAGE (bp_app_). The ratio of the apparent size to the actual sequence size is also presented (bp_app_/bp_actual_). Band 1 is the species with highest mobility and is assumed to be the normal B-DNA (see Figure 2A). Additional bands are numbered in decreasing order of mobility. The 200 repeat PCR product generated a series of poorly resolved products and only a single non-B-DNA species has been defined*.

Assuming that the fast mobility band represents linear duplex B-DNA (Pearson and Sinden, 1996; Pearson et al., 1998), a densitometric analysis was performed in an attempt to quantify the propensity of different repeat sizes to adopt alternative S-DNA (Figure 2D). These data revealed that the proportion of linear duplex DNA decreases as the repeat gets longer, reaching a plateau around 50 CAG•CTG repeat units. Non-linear regression analysis revealed that the number of repeats in each fragment explained a very high proportion of the variation in the fraction of non-B DNA detected in both agarose (*r^2^* = 0.99, *p* = 0.02) and native polyacrylamide gels (*r*^2^ = 0.85, *p* = 0.03). This observation confirms that lengthening of the repeat tract enhances the propensity to form S-DNA structures, as described in earlier reports (Pearson et al., 1998).

### Confirmation of S-DNA Folding within CAG•CTG Sequences

**Figure 3:**
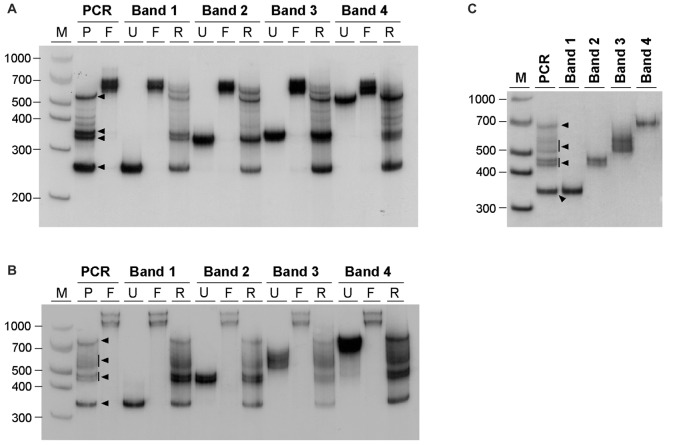
**Generation of multiple alternative DNA structures following re-annealing of gel purified CAG•CTG repeat sequence isomers**. PCR products containing either **(A)** 22 or **(B)** 56 CAG•CTG repeats were amplified with oligonucleotide primers DM-A and DM-DR. DNA was extracted from single bands following native PAGE (bands 1–4, black arrowheads) and re-electrophoresed (lanes marked U, un-denatured). Duplex DNA samples were melted at 100°C in the presence of 40% (v/v) formamide, revealing single-strand DNA bands of low mobility (lanes marked F, formamide). Alternatively, DNA samples were denatured at 100°C, and subsequently re-annealed by a slow decrease in temperature over 3 h (lanes marked R, re-annealed), producing a complex pattern of bands, previously observed in the original PCR product. **(C)** Incubation of isolated DNA species with 500 nM ethidium bromide prior to re-electrophoresis did not change the mobility of individual bands extracted from PCR products with 56 CAG•CTG repeats. The molecular size markers in bp (M) are shown.

The similarity between the electrophoretic profiles observed when PCR products were electrophoresed in native polyacrylamide gels and previous reports of S-DNA (Pearson and Sinden, 1996; Pearson et al., 1998) is a strong indication that slipped-stranded non-B-DNA structures were already present in the PCR products generated. Further experimental support was obtained by melting and re-annealing DNA purified from single bands collected from non-denaturing polyacrylamide gels. Individual bands were isolated from two PCR products containing either 22 or 56 CAG•CTG repeats, since they gave rise to discrete sharp individual bands, easily purified from the gel (Figures 3A,B). For each DNA species derived from a single band a denaturation and a re-annealing analysis was performed, followed by native PAGE. All gel-purified DNA species migrated as single bands (Figures 3A,B, lanes U), confirming their high stability throughout the elution and precipitation procedures. DNA melting in the presence of formamide resulted in the detection of two DNA bands with very low mobility (Figures 3A,B, lanes F), which correspond either to linear single-stranded DNA or to an aggregation of DNA formed during denaturation. In contrast, incubation with ethidium bromide prior to PAGE did not change the migration pattern of gel purified DNA species (Figure 3C), confirming that the intercalating chemical is unable to interconvert structural DNA isoforms. The full set of anomalously migrating bands observed for the original PCR product was restored following the re-annealing of individual isolated bands (Figures 3A,B, lanes R). The observation that the re-annealing protocol resulted in a pattern of products that was very similar to that previously observed in the original PCR products, further confirmed that the slow migrating DNA species detected correspond to S-DNA formed during the re-hybridization of DNA strands.

### Generation of Alternative Structures by PCR Amplification

**Figure 4:**
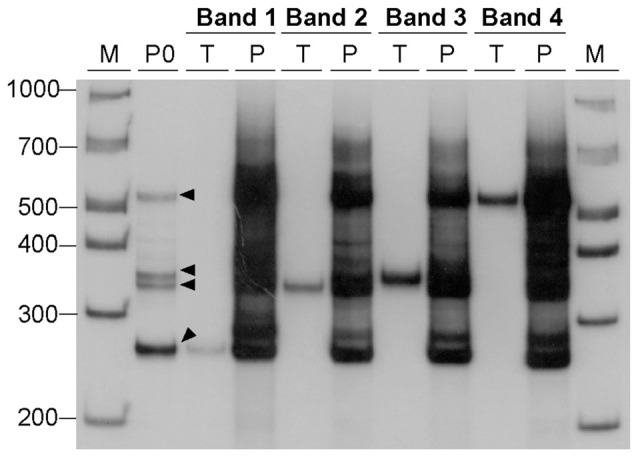
**Generation of alternative DNA structures by PCR amplification**. DNA samples, containing 22 CAG•CTG repeat units, were eluted from four bands (bands 1–4, black arrowheads) excised from a native 8% (w/v) polyacrylamide gel under UV light, and used as templates for a second PCR amplification. Each DNA sample was re-electrophoresed before (lanes marked T, template) and after the second PCR amplification (lanes marked P, PCR product). The amplification resulted in a complex pattern of bands very similar to that observed in the original PCR product (lane marked P0) from which the template was extracted. The molecular size markers in bp (M) are shown.

Given our hypothesis that the additional bands observed by agarose gel electrophoresis and native PAGE represent S-DNA conformations adopted by CAG•CTG repetitive structures, our results indicate that non-B-DNA structures are generated under standard PCR cycling conditions (<30 amplification cycles). To confirm that PCR amplification is sufficient to produce alternative DNA structures, dilutions of the gel-purified DNA samples derived from single bands and carrying 22 CAG•CTG repeats were used as template in a second amplification reaction, with the same oligonucleotide primers. The DNA samples were analyzed by native PAGE before and after the second round of PCR amplification (Figure 4). The products generated by the second amplification reaction migrated as a complex pattern of heterogeneous bands, exhibiting electrophoretic profiles very similar to those detected in the original PCR product, as well as following the re-annealing protocol. In summary, the amplification of DNA samples, derived from the human *DM1* locus by standard PCR techniques generates a complex mixture of alternative DNA conformations, which are interconvertible following denaturation and re-annealing protocols.

## Discussion

The molecular mechanism(s) of trinucleotide repeat size mutation associated with human disorders is likely to involve the unique structural properties associated with simple trinucleotide repeat DNA sequences. The formation of DNA structures such as hairpins and slipped-stranded DNA may affect normal DNA metabolism, and lead to the unusual biology associated with triplet repeats (Mitas, 1997; Sinden et al., 2002; Mirkin, 2006). We have developed a simple agarose gel-based method to identify the presence of alternative non-B-DNA structures adopted by double-stranded DNA sequences containing more than five trinucleotide CAG•CTG repeat units. Slow migrating DNA species, representing unorthodox DNA conformations adopted by CAG•CTG-containing molecules, are readily detected by agarose gel electrophoresis in the absence of ethidium bromide. In the presence of ethidium bromide, the same products collapse and migrate as a single DNA species. Importantly, we have shown that high levels of such alternative structures are formed by low number of cycles (<30) during PCR amplification of low template DNA concentrations, as well as standard PCR amplification of thousands of genomic DNA template sequences. Therefore it is strongly recommended that accurate sizing of CAG•CTG repeat-containing PCR products by agarose gel electrophoresis is performed in the presence of ethidium bromide. The omission of ethidium bromide from gels and running buffers results in the detection of additional DNA species, which may be incorrectly classified as mutant alleles, leading to an over-estimation of mutation frequencies and/or misdiagnosis of affected status.

The reduced gel mobility of the additional bands observed in the absence of ethidium bromide is consistent with that expected for DNA containing bends introduced from the three- and four-way junctions generated by the looped-out strands characteristic of slipped-stranded DNA structures (Pearson et al., 1998). Ethidium bromide intercalates into DNA and is capable of untwisting non-repetitive DNA sequences, destabilizing the fine stacking interactions (Brukner et al., 1997; Hayashi and Harada, 2007; Lipfert et al., 2010) and increasing their mobility through agarose gels (Brukner et al., 1997). Therefore, when added to the agarose gel and electrophoresis buffer, ethidium bromide most likely modifies the migration of structural isoforms of CAG•CTG repetitive sequences by relaxing the duplex and increasing the flexibility of the bend. Interestingly, ethidium bromide does not modify the mobility of structural isoforms through native polyacrylamide gels, in which normal B-DNA triplet repeats tend to show increased mobility (Chastain et al., 1995; Chastain and Sinden, 1998; Williams et al., 1999). The higher resolution of polyacrylamide gels, may explain the greater complexity of the electrophoretic profiles and the higher levels of non-B-DNA species detected relative to agarose gel electrophoresis. It appears that agarose gels are only capable of resolving B-DNA from a heterogeneous subset of non-B-DNA species, whereas polyacrylamide gels resolve different forms of non-B-DNA molecules.

**Figure 5:**
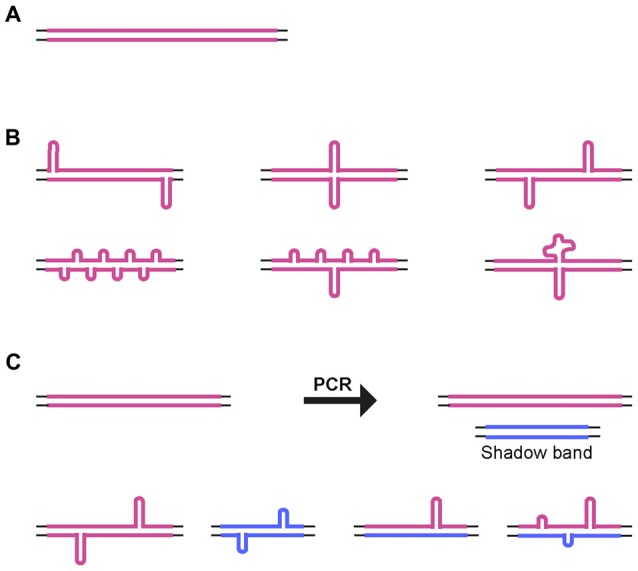
**Alternative DNA structures. (A)** Schematic representation of homoduplex B-DNA. **(B)** Models of possible structural isomers of S-DNA generated by denaturation and out-of-register re-annealing of complementary trinucleotide repeat strands. Looped-out regions may be of variable size and/or number, and they may be positioned throughout the repeat tract. **(C)** Generation of small size repeat variants by DNA polymerase slippage during PCR (shadow bands). Denaturation and out-of-register re-annealing generate homo- and heteroduplex double-strand DNA structures. Thin lines represent the unique sequence flanking DNA. Thick colored lines represent complementary CTG and CAG expanded DNA sequences.

For all of the CAG•CTG repeat PCR products analyzed, a significant proportion of the products migrated in native polyacrylamide gels as a broad distribution of species with mobilities equivalent to more than twice their actual size. Previous electron microscopy analyses have shown that two complementary strands with the same number of repeats paired in an out-of-register fashion form homoduplex S-DNA secondary structures at random locations throughout the repeat tract, giving rise to a slow migrating and heterogeneous population of products (Pearson et al., 1998; Figure 5). Nevertheless, the various structural isomers detected did not result in an evenly distributed smear of products: certain mobility variants were favored and observed as discrete bands, indicating that many isomers have near identical electrophoretic mobilities, as previously suggested (Pearson and Sinden, 1996; Pearson et al., 1998, 2002). Given the possibility of DNA polymerase slippage and the generation of shadow bands during PCR amplification of trinucleotide repeat sequences (Wu et al., 1998; Hunter et al., 2005; Mulero et al., 2006; Olejniczak and Krzyzosiak, 2006; Blanco et al., 2008; Gibb et al., 2009), heteroduplex double-strand DNA products might also be formed. Therefore, at least some of the DNA species detected by native PAGE may correspond to slipped-stranded intermediate DNA structures (SI-DNA), formed by the re-annealing of single-stranded trinucleotide sequences with different repeat numbers (Figure 5).

Interestingly, short repeat lengths that are not associated with disease (5 and 22 CAG•CTG repeat units) also exhibited the potential to generate stable alternative structures *in vitro*. Thus, the ability to fold into non-B-DNA conformations *per se* does not distinguish long unstable pathogenic repeats from short stable repeats. Importantly, longer disease-associated repeats display, however, a higher propensity to form slipped-stranded DNA structures, as estimated by the percentage of linear B-DNA detected by native PAGE. Furthermore, it was previously speculated that long repeat stretches form alternative structures with notably longer lifetimes (Gacy et al., 1995). Long-lived stable secondary structures might be viewed as substrates for a mechanism of repeat size expansion that involves slipped-stranded DNA intermediates that are recognized and repaired by the DNA mismatch repair pathway (Gomes-Pereira and Monckton, 2006; López Castel et al., 2010; McMurray, 2010), thereby explaining why expansion occurs with higher frequency at long repeats, but not at short repeats. Mismatch recognition is mediated by DNA bending (Wang et al., 2003; Sass et al., 2010; Hura et al., 2013). Thus, compounds such as ethidium bromide that intercalate into DNA and modify bending properties, might be expected to alter mismatch recognition and trinucleotide repeat dynamics. The stabilization of expanded CAG•CTG repeats in a mouse cell culture system of unstable DNA by ethidium bromide (Gomes-Pereira and Monckton, 2004) might therefore be, at least in part, a consequence of an effect of the intercalating chemical on DNA structural biology. A greater understanding of triplet repeat DNA structures and the molecular mechanisms for triplet repeat expansion may facilitate the development of new routes to therapy based upon modifying DNA dynamics.

The assay described herein opens new avenues for the study of alternative DNA structures adopted by trinucleotide repeat sequences *in vivo*. Agarose gel electrophoresis of digested genomic CAG•CTG-containing DNA in the absence of ethidium bromide might reveal non-B-DNA structures formed *in vivo* upon detection by Southern blot hybridization. Indeed, it is possible that the presence/absence of ethidium bromide in agarose gels might be affecting the electrophoretic migration and smears currently detected in diagnostic Southern blot hybridizations of genomic DNA. In line with this hypothesis, the unique interaction between ethidium bromide, genomic trinucleotide repeat sequences and their electrophoretic mobility has been reported for CGG•CCG repeat expansions: ethidium bromide reduces the mobility of genomic DNA fragments derived from the fragile X syndrome locus (Cummins, 1997; Nolin et al., 2008). The opposing effect of the intercalating dye on the mobility of genomic CGG•CCG DNA and PCR-generated CAG•CTG products remains unexplained. It may depend on the nucleotide sequence of the repeat itself, the different types of non-B-DNA structures adopted by CGG•CCG or CAG•CTG repeats, their chemical interaction with ethidium bromide or the structural changes specifically introduced by PCR amplification. Following on from these results, and to avoid the possibility of incorrectly sizing expanded alleles, the inclusion of ethidium bromide in diagnosis and research procedures must be carefully considered. While the elimination of ethidium bromide from fragile X Southern analysis is suggested (Cummins, 1997; Nolin et al., 2008), we propose that all diagnostic and research procedures based upon agarose gel electrophoresis of CAG•CTG products generated by standard PCR should incorporate ethidium bromide in the gel and running buffer.

## Author Contributions

MG-P: experimental work, data acquisition and preparation of figures. MG-P and DGM: study design/interpretation, data analysis and manuscript preparation.

## Conflict of Interest Statement

The authors declare that the research was conducted in the absence of any commercial or financial relationships that could be construed as a potential conflict of interest.
